# Effect of Grain Size on Nanometric Cutting of Polycrystalline Silicon via Molecular Dynamics Simulation

**DOI:** 10.3390/mi15060767

**Published:** 2024-06-08

**Authors:** Wen Guo, Qiuyue Yu, Guoyan Wang, Shuming Fu, Changlin Liu, Xiao Chen

**Affiliations:** 1 Optical Ultra Precision Machining Technology Innovation Center, Beijing Institute of Space Mechanics and Electricity, Beijing 100094, China; 18612996358@139.com (W.G.); yuqiuyue1231@163.com (Q.Y.); wangyaya8@163.com (G.W.); fusm508@163.com (S.F.); 2 State Key Laboratory of Ultra-Precision Machining Technology, Department of Industrial and Systems Engineering, The Hong Kong Polytechnic University, Hong Kong 999077, China; 3 Hubei Key Laboratory of Modern Manufacturing Quality Engineering, School of Mechanical Engineering, Hubei University of Technology, Wuhan 430068, China; chenxiao1987jz@163.com

**Keywords:** nanometric cutting, molecular dynamics simulation, polycrystalline silicon, surface formation, subsurface damage

## Abstract

The grain size effect is an important factor in determining the material removal behavior of polycrystalline silicon (p-Si). In the present study, to improve the understanding of nanoscale machining of p-Si, we performed molecular dynamics simulation of nanometric cutting on a p-Si workpiece and discussed the grain size effect on material removal behavior and subsurface damage formation. The simulation results indicate that when cutting on the polycrystal workpiece, the material removal process becomes unstable compared with single crystals. Higher removal efficiency, less elastic recovery and higher frictional coefficient are observed as the average grain size decreases. In the subsurface workpiece, when the grain size decreases, slip along grain boundaries merges as a nonnegligible process of the plastic deformation and suppresses the elastic deformation ahead of the cutting tool. It is also revealed that when cutting on a polycrystal workpiece with smaller grains, the average stress decreases while the workpiece temperature increases due to the impediment of heat transfer by grain boundaries. These results could provide a fundamental understanding in the material deformation mechanism of p-Si during nanoscale machining.

## 1. Introduction

Polycrystalline silicon (p-Si) is an essential material in the solar photovoltaic and electronics industries due to its superiorities in stability and manufacturing costs [1]. With rapid improvement of the device performance in these applications, the requirement for components with high surface quality is continuously increasing. To meet this demand, nanoscale machining technologies, including nanometric cutting and grinding, have gradually become a topic of great interest in advanced manufacturing. Their feasibility in fabrication of surfaces with nanoscale roughness and low subsurface damage on various materials has been verified [2,3,4]. During nanoscale machining, since the material removal thickness ranges from tens to hundreds of nanometers, which is comparable to the cutting tool edge, the wear mechanism of grains and removal behavior of the workpiece material can be distinct with conventional machining [5,6]. For polycrystalline materials, characteristics of workpiece microstructures like crystal orientation and grain boundaries (GBs) could have a nonnegligible influence on the deformation behavior at nanoscale. Therefore, it is critical to explore the material removal mechanism on an atomic scope to improve understanding in the nanoscale machining mechanism of p-Si.

During the nanoscale machining process, deformation of the workpiece material is usually difficult to observe and measure by experiments. In recent years, molecular dynamics (MD) simulation has gradually become an effective method to reveal the mechanisms in nanoscale machining [7,8]. Based on this method, the structural evolution of workpiece materials, including dislocation [9] and phase transition [10], can be investigated at atomic level. For instance, Fang et al. [11] established an MD model of nanometric cutting to investigate the material removal behavior of single-crystal Si. They found that ductile mode removal of single-crystal Si is dominated by extrusion of the disordered atoms when the material removal thickness decreases to nanoscale. In another study by Wang et al. [12], they suggested that the contact-induced amorphization dominates the extrusion process, while strain-induced amorphization is responsible for shearing removal of single-crystal Si. Lai et al. [13] conducted MD simulation of nanometric cutting on single-crystal Ge. They built a partially overlapped cutting model to study the chip side flow of Ge atoms with different machining parameters [14], which is more representative of the actual machining environment. Goel et al. [15] used MD method to investigate the deformation mechanism of single-crystal 3C-SiC in nanometric cutting. They revealed that the crystal anisotropy of the workpiece plays an important role in determining the dominant material removal mechanism. Meng et al. [16] studied the influence of strain rate and temperature on the removal behavior and wear mechanism of 3C-SiC. They suggested that the abrasive wear behavior is caused by the coupling action of impact effect and grinding heat. In other research, Zhou et al. [17] revealed that the diamond abrasives are worn out through a combination of thermochemical wear, graphitization wear and abrasive wear during nanoscale machining. Furthermore, Tian et al. [18] established MD models of nanoscale indentation and scratching to explore the deformation mechanisms of single-crystal 4H- and 6H-SiC. Their results indicated that the amorphous phase on the machined surface can be generated due to the slip motion.

In addition to single crystals, the machining mechanism of polycrystalline materials is more complicated as it composes grains and GBs with random orientations. Zhao et al. [19] conducted an MD simulation of nanometric cutting on polycrystalline Cu with hexagon grains. Their results indicated that periodic formation and annihilation of the sub-grains can be caused by extrusion between the GBs and cutting tool. Fan et al. [20] performed a nano-scratching simulation of polycrystalline gallium arsenide (GaAs) with grains of random size and orientation. They revealed that the deformation of polycrystalline GaAs is accompanied by dislocation nucleation in the GBs. Liu et al. [21] used the same method to investigate the material removal behavior of polycrystalline 3C-SiC and found that the microstructures, including grain geometry and GBs, could influence the internal stress and suppress the amorphization process. In other research, Zhao et al. [22] discussed the material removal behavior of polycrystalline 3C-SiC in nanometric cutting and suggested that the grain size has a great influence on the brittle-to-ductile transition. Specific to p-Si, Goel et al. [23] built an MD model for nanometric cutting of p-Si and revealed that the existence of GBs could facilitate the amorphization of Si and cause irregular groove patterns on the machined surface, which is apparently different from single crystals. Liu et al. [24] suggested that during nanometric cutting of p-Si, fracture of the polycrystalline workpiece mainly includes intra-granular and inter-granular modes, which could cause voids and cracks on the machined surface. The abovementioned research has expanded the knowledge on the nanoscale machining mechanism of p-Si. However, as an important factor in determining the machinability, the grain size effect on machining mechanism of p-Si has not been revealed, and more exploration of the intricate relationship between the cutting tool and workpiece with different grain sizes are required to gain a more complete picture of the machining mechanism of p-Si.

Therefore, in the present study, we used MD simulation to explore the machining mechanism of p-Si during nanometric cutting. The effect of grain size on the material removal behavior and subsurface damage evolution was discussed. The large-scale atomic/molecular massively parallel simulator (LAMMPS) [25] and Open Visualization Tool (OVITO) [26] were employed to conduct the simulation and visualize the output data. The results in this study could provide a fundamental understanding of the material deformation of p-Si during nanoscale machining and guide the improvement of machining performance for other polycrystalline materials.

## 2. Methodology

**Figure 1:**
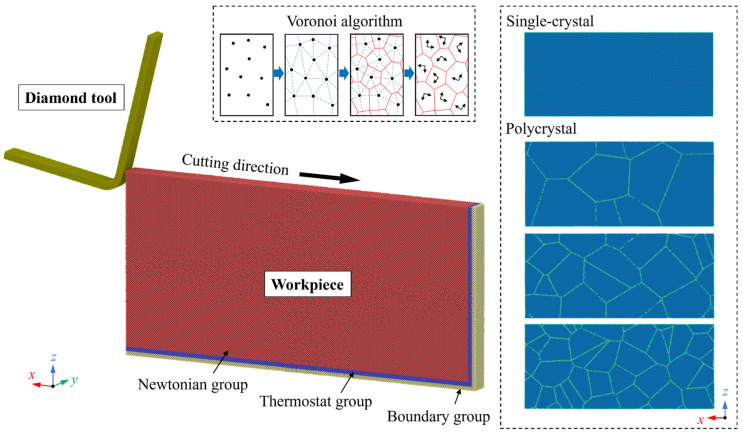
The MD model for nanometric cutting simulation of polycrystalline Si.

Figure 1 displays the MD model for nanometric cutting simulation, in which common neighbor analysis (CNA) [27] was used to determine the crystal structure of Si atoms. The workpiece is set as deformable while the diamond tool is considered as a rigid body. The polycrystal structure of the p-Si workpiece was created by Atomsk [28] based on the Voronoi algorithm [29,30]. In this algorithm, the polycrystal structure is generated by combining the normal of the lines that link the random points to form grains with seeds that have random crystal orientation, while the (010) plane is fixed on the x–z plane. To explore the grain size effect on machining mechanism, cutting simulation was conducted on workpieces with different number of grains. The average diameter of the crystal grain in the polycrystal workpiece is set as 20.08 nm, 10.04 nm, and 5.60 nm while cutting simulation of a single-crystal workpiece was conducted in the [100] (001) direction with the same parameters for comparison. Following the classic setup in MD simulation of nanometric cutting [31], the workpiece atoms are divided into boundary group, thermostat group, and Newtonian group. The fixed boundary condition is applied in the *x* and *z* directions to limit the simulation size, while the periodic boundary condition is applied along the *y* direction to eliminate the size effect. The workpiece atoms were equilibrated to release internal stress and thermal expansion at 300 K and 0 PGa for 150 ps before the cutting simulation. Table 1 shows the detailed simulation parameters.

In MD simulation, adopting a reliable potential function for the atomic interactions is critical to ensure the accuracy of the results. In the present study, the analytical bond order potential (ABOP) [32] is used to describe the atomic interactions in the workpiece (Si-Si) and cutting tool (C-C), as it demonstrates a good approximation in mechanical properties of diamond and diamond-like structures [33,34]. The interaction between workpiece and tool (Si-C) is described by the Morse potential, which has been verified as an efficient and accurate selection [35,36]. The function of the Morse potential can be expressed as:

(1) $$E_{\mathrm{Si}\text{-}C}(r_{\mathrm{ij}})=D_{M}[e^{-2a(r_{\mathrm{ij}}\text{-}\;r_{M})}-2e^{-a(r_{\mathrm{ij}}\text{-}\;r_{M})}]$$

where *D_M_*, *a*, and *r_M_* are the cohesion energy, modulus of elasticity, and the equilibrium distance between atoms. According to previous research [37,38], the parameters are set as: *D_M_* = 0.435 eV, *a* = 46.487 nm^−1^, *r_M_* = 0.19475 nm.

## 3. Results and Discussion

### 3.1. Material Removal Behavior

#### 3.1.1. Atomic Flow

**Figure 2:**
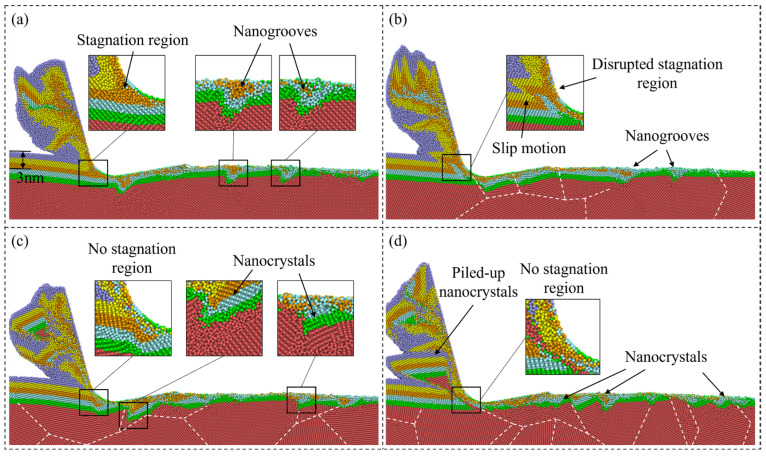
Illustration of atoms flow in nanometric cutting. (**a**) Snapshot of the single-crystal workpiece. (**b**–**d**) Snapshots of polycrystal workpieces with average grain diameters of 20.08 nm, 10.04 nm, and 5.60 nm.

In nanometric cutting, the material removal behavior is greatly determined by the atomic flow in the deformation region of the workpiece. For single-crystal Si, a stagnation region is usually observed ahead of the cutting tool edge, which is an area where the workpiece atoms are relatively stationary in relation to the cutting tool. The atoms beneath the stagnation region are compressed into the subsurface workpiece, while those above the stagnation region would be piled up into chips and removed. Figure 2 presents the atomic flow of workpiece material during nanometric cutting. To visualize the deformation behavior, atoms in the deformation zone are divided into layers with different colors. As shown in Figure 2a, a clear stagnation region is formed at a depth of about 3 nm when cutting on the single-crystal workpiece. Similar to the experimental observation [39], arrays of nanogrooves are formed on the machined surface and extend into the workpiece at an angle of −45° to the cutting direction, which is related to accumulation and release of the compression-induced strain energy and slip motion of the crystal lattice [23]. When cutting on the polycrystal workpiece, atomic flow of the workpiece atoms is affected by GBs and sliding of crystal grains. As the grain size decreases, the stagnation region becomes less apparent as piling up of the grains could interrupt the flow of the disordered atoms, which indicates a less stable material removal process compared with single crystals. Besides, more nanogrooves are observed at GBs than the crystal surface since the generated disordered atoms are squeezed into boundaries. Meanwhile, more nanocrystals can be generated and compressed into GBs with disordered atoms, as shown in Figure 2c,d.

**Figure 3:**
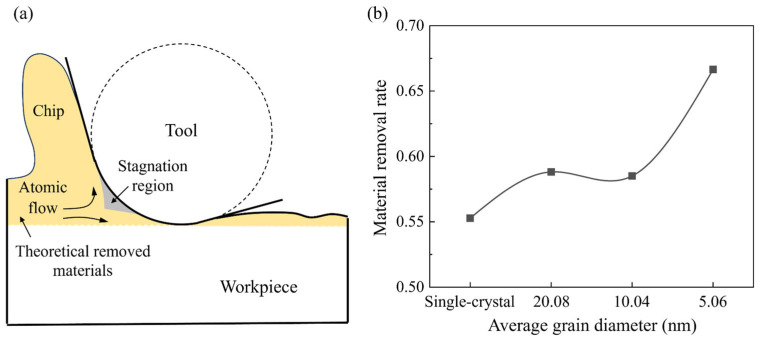
Material removal rate in nanometric cutting. (**a**) Illustration of the actual material removal process in nanometric cutting. (**b**) The calculated material removal rate when cutting on single-crystal and polycrystal workpieces.

**Figure 4:**
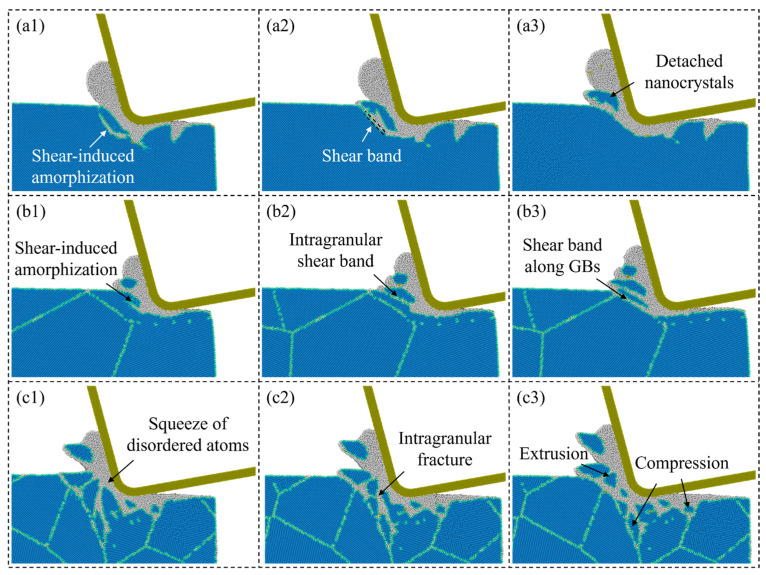
Snapshots of the chip formation during cutting on: (**a1**–**a3**) single-crystal workpiece, (**b1**–**b3**) and (**c1**–**c3**): polycrystal workpieces with average grain diameters of 20.08 nm and 5.60 nm.

Due to the downward compression of the workpiece atoms beneath the stagnation region, the actual material removed is usually less than the theoretical situation, as shown in Figure 3a. To measure the grain size effect on removal behavior, the material removal rate is calculated when the cutting distance reaches 70 nm, which is defined as the ratio between the number of the actual and theoretical removed atoms, as shown in Figure 3b. It is observed that the material removal rate for polycrystal workpiece is higher than single crystals and apparently rises as the grain size decreases. In nanometric cutting of Si, the extrusion-dominated removal process usually has a lower material removal efficiency than the shear removal mode since comparable materials are compressed downward into the subsurface workpiece. While when cutting on p-Si, the workpiece material can be removed by the slip motion of grains along the GBs and nanocrystals can be piled up into chips as the grain size decreases, which is advantageous to reduce the downward compression and improve the material removal rate. Figure 4 presents snapshots of chip formation during cutting on single-crystal and polycrystal workpieces where the Si atoms are colored by crystal structure determined by CNA. Blue atoms represent the cubic diamond structure while the disordered atoms are colored in white, which mainly contains amorphous phase, metallic phase, and other defective atoms [40]. These structures are unstable and most of them would transform into disordered structures (mainly amorphous phase) after cutting [31]. When cutting on the single-crystal workpiece, as shown in Figure 4(a1,a2), a shear band can be formed by the shear-induced amorphization in the deformation region, causing detachment and extrusion of the nanocrystals into chips, while for the polycrystal workpiece, as shown in Figure 4(b1,b2), the shear-induced amorphization and intra-granular shear band can be observed when the cutting tool moves into the crystal grains. The formation of the shear band is also affected by GBs, which causes slide of the nanocrystals into chips, as shown in Figure 4(b3). When the grain size decreases, squeezing of the disordered atoms into GBs can be apparent and intra-granular facture is observed ahead of the cutting tool, which causes massive nanocrystals in the deformation region. With further advances of the cutting tool, the nanocrystals can be compressed into GBs or extruded into chips, as shown in Figure 4(c3).

#### 3.1.2. Surface Morphology

**Figure 5:**
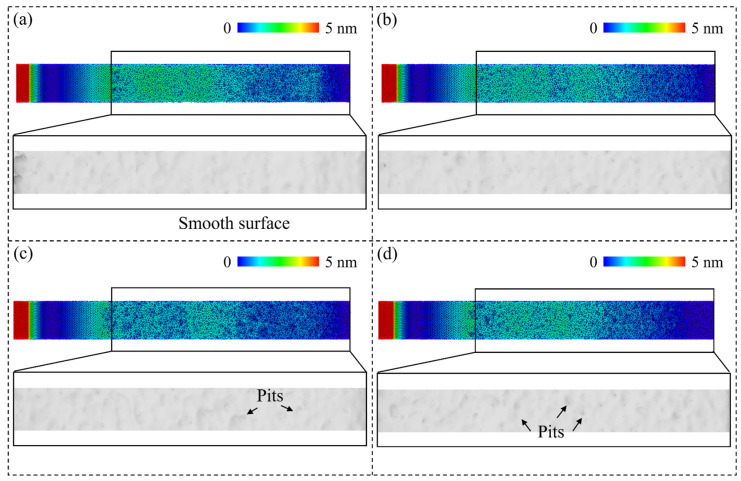
The height distribution of workpiece atoms and constructed surface of: (**a**) single-crystal workpiece. (**b**–**d**) Polycrystal workpieces with average grain diameters of 20.08 nm, 10.04 nm, and 5.60 nm.

**Figure 6:**
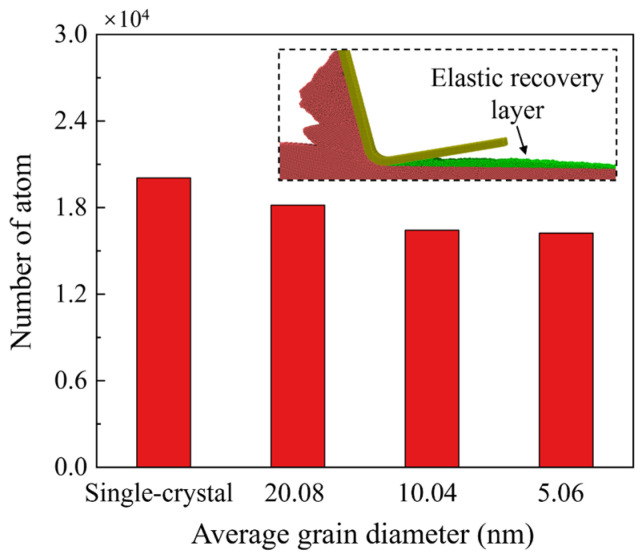
Number of workpiece atoms in the elastic recovery layer as the cutting distance reaches 70 nm.

As mentioned above, the workpiece material beneath the stagnation region is compressed into the workpiece. After machining, these materials tend to release the strain energy by returning to their balanced position. The machining-induced metastable Si phases in the deformation region experience relaxation and phase transition to a stable state with an obvious volume expansion during unloading [41], which can be identified as the elastic recovery or swelling effect. Figure 5 shows the height distribution of workpiece atoms and constructed machined surface, which is generated by the alpha-shaping method [42]. It is observed that after the initial contact, elastic recovery gradually becomes apparent on the machined surface due to the increased compression in the deformation region. Different from the smooth surface and continuous elastic recovery layer when cutting on single crystals, the generated elastic recovery layer on polycrystal workpiece is irregular and more pits are observed on the machined surface as the grain size decreases, which can be attributed to the unstable atomic flow induced by slip motion along GBs. Furthermore, Figure 6 presents the number of the atoms in the elastic recovery layer when the cutting distance reaches 70 nm. As the grain size decreases, a decrease in atoms in the elastic recovery layer is observed, since the accumulated strain energy during relaxation and volume expansion of the metastable phases can be dissipated through GBs when cutting on a polycrystal workpiece.

#### 3.1.3. Cutting Force

**Figure 7:**
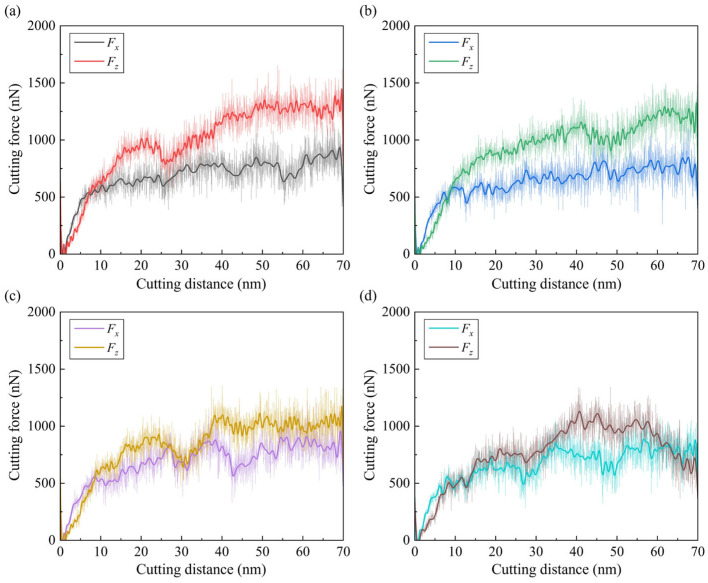
Variation in transient cutting forces as a function of the cutting distance for: (**a**) single-crystal workpiece. (**b**–**d**) Polycrystal workpieces with average grain diameters of 20.08 nm, 10.04 nm, and 5.60 nm.

**Figure 8:**
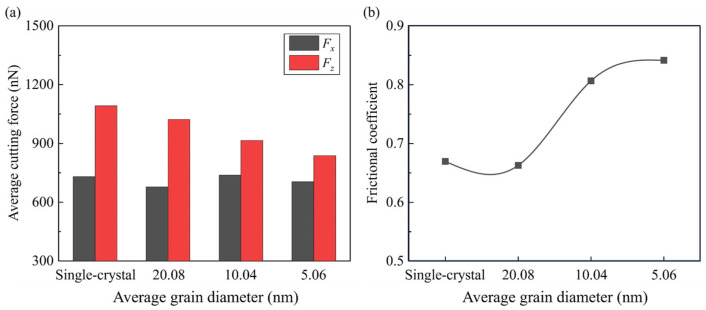
(**a**) The average cutting forces. (**b**) Frictional coefficient for different simulation cases.

In nanometric cutting, variation in the cutting forces is an important indicator in identifying the material removal behavior. Figure 7 shows the transient tangential force (*F_x_*) and nominal force (*F_z_*) as a function of the cutting distance during the simulation. Due to the increase in the contact area between tool and workpiece, the cutting forces grow quickly in the initial stage and then fluctuate at a relatively stable value. It is observed that the increase in tangential force is more obvious than the nominal force, which can be attributed to the rise in material load in front of the tool rake face. Due to the negative tool rake angle, the stabilized nominal force is apparently larger than the tangential force, which illustrates strong friction between the tool and workpiece surface [39]. Furthermore, as the grain size decreases, the fluctuation in cutting forces becomes more obvious, since the existence of grains and boundaries obviously affects the deformation and piling up of workpiece material. The average cutting forces at the stable stage (as the cutting distance ranges from 10 nm to 70 nm) are shown in Figure 8a. It is observed that the nominal force for the polycrystal workpiece is smaller than single crystals and decreases as the grain size decreases. This variation indicates that increasing that the density of GBs tends to have a more apparent influence in releasing the downward compression than the resistance of cutting tool motion, which is more sensitive to the material load. Therefore, the average frictional coefficient, which is defined as the averaged *F_x_*/*F_z_* in the stable stage, is increased when the average grain size decreases, as shown in Figure 8b.

### 3.2. Subsurface Damage Formation

#### 3.2.1. Plastic Deformation

**Figure 9:**
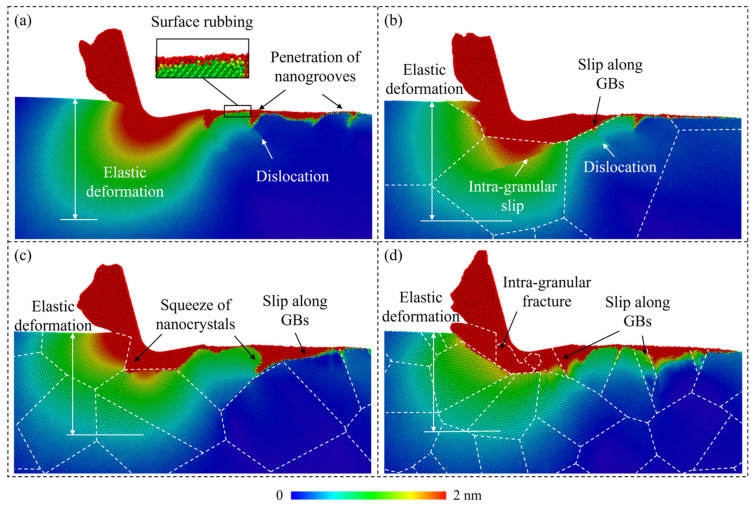
Displacement magnitude of workpiece atoms when cutting on: (**a**) Single-crystal workpiece. (**b**–**d**) Polycrystal workpieces with average grain diameters of 20.08 nm, 10.04 nm, and 5.60 nm.

For a polycrystal workpiece, the plastic deformation in the workpiece is greatly influenced by subsurface microstructures, including grains and boundaries. In MD simulation, deformation of the subsurface workpiece can be illustrated by analyzing the displacement of workpiece atoms after machining. When the workpiece material undergoes elastic deformation, atoms would depart from their balanced positions and their displacement magnitude would vary constantly with their neighbors, while when plastic deformation occurs, the crystal structure would be destroyed and there would be a clear break in the displacement magnitude distribution. Figure 9 presents the distribution of the displacement magnitude of workpiece atoms as the cutting distance reaches 60 nm. When cutting on the single-crystal workpiece, an apparent elastic deformation region is formed ahead of the cutting tool, while the plastic deformation pattern on the machined surface mainly includes surface rubbing, penetration of nanogrooves, and dislocation propagation, as shown in Figure 9a. While when cutting on the p-Si workpiece, slip along the GBs merges as a nonnegligible process of the plastic deformation in the workpiece. With the decrease in grain size, the elastic deformation ahead of the cutting tool can be less apparent as the strain energy is dissipated by enhanced slip motion. Meanwhile, formation and squeeze of nanocrystals becomes more apparent due to the intra-granular facture of crystal grains.

#### 3.2.2. Phase Transition

**Figure 10:**
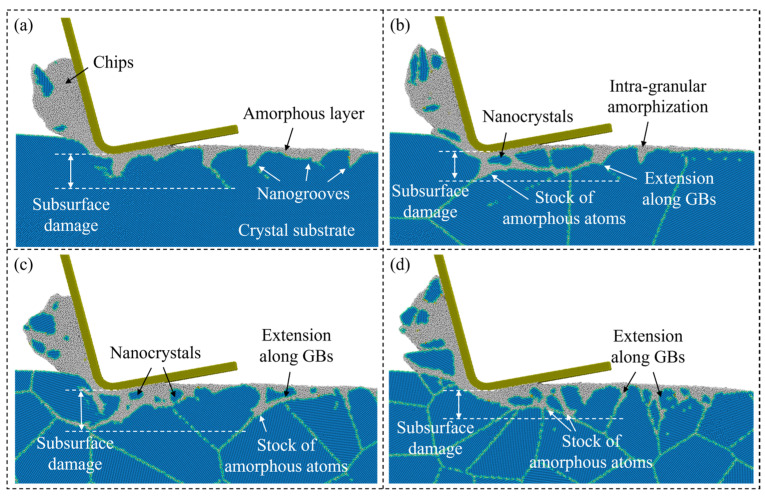
Crystal structure of the Si atoms when cutting on: (**a**) single-crystal workpiece. (**b**–**d**) Polycrystal workpieces with average grain diameters of 20.08 nm, 10.04 nm, and 5.60 nm.

**Figure 11:**
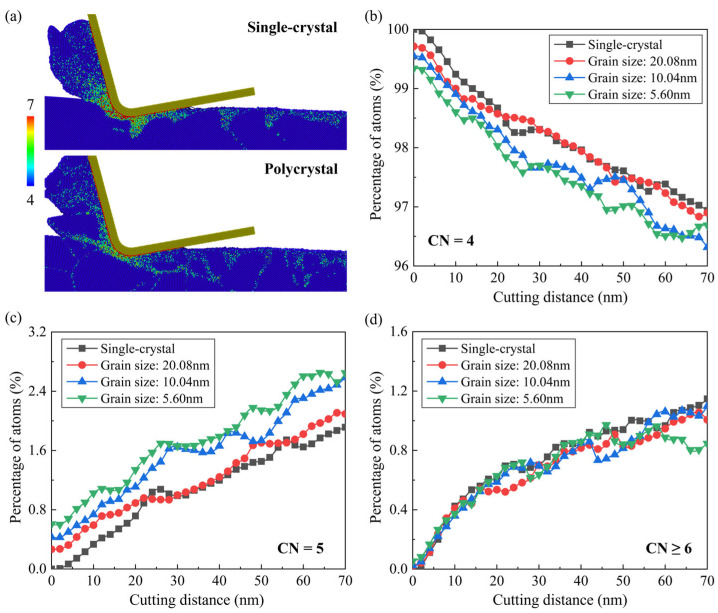
Coordination numbers in workpiece atoms. (**a**) Snapshots of the workpiece atoms colored by coordination number in single-crystal and polycrystal workpieces with average grain diameter of 5.60 nm. (**b**–**d**) Variation in atoms with different coordination numbers during the cutting process.

In nanometric cutting of Si, the high-pressure phase transition (HPPT) to amorphous and metastable phases is thought to be a critical mechanism of ductile deformation. It significantly affects the generation of subsurface damage during machining as well as the brittle-to-ductile transition [43,44]. Figure 10 shows the crystal structure of the subsurface workpiece as the cutting distance reaches 70 nm, where the Si atoms are colored by CNA. When cutting on a single-crystal workpiece, an amorphous layer with nanogrooves is formed on the machined surface due to HPPT and relaxation of the metastable phases, while when cutting on the polycrystal workpiece, extension of the amorphous atoms is obviously affected by the microstructures in the workpiece. The generated amorphous atoms can be squeezed into GBs along with the slip motion of crystal grains. As the grain size decreases, inter-granular amorphization becomes more apparent than the intra-granular amorphization and extension of the amorphous atoms can be promoted, causing more nanocrystals on the machined surface. The stock of the amorphous atoms can be observed at triple junctions where the extension of amorphous phases intersects, which could lead to fracture of the workpiece [24]. In addition to the subsurface damage extension, transition of the atoms into structures with high coordination number (CN), such as β-Sn (CN = 6) and Bct5 (CN = 5), phases can be observed during HPPT in the deformation region. Figure 11a shows snapshots of workpiece atoms colored by CN when the cutting distance reaches 70 nm in single-crystal and polycrystal workpieces with average grain diameter of 5.60 nm. As the cutting tool advances, the generation of the over-coordinated atoms (CN > 4) coincides with the deformation layer expanding ahead of the cutting direction. Most atoms with high coordination numbers (CN ≥ 6) are found close to the tool edge, while some 5-coordinated atoms remain on the machined surface after cutting. For a polycrystal workpiece, the concentration of atoms with high coordination numbers (CN ≥ 6) is less apparent near the cutting tool edge compared with that in single crystals. Some 5-coordinated atoms are observed in GBs, indicating that the structure distortion could be more obvious near the boundaries after cutting. The variation in atoms with different CNs in a workpiece is present in Figure 11b–d. It is observed that the number of 4-coordinated atoms decreases and the over-coordinated atoms gradually increase as the cutting tool advances. For the polycrystal workpiece, more 5-coordinated atoms are generated, especially when the grain size decreases. The number of atoms with high coordination numbers (CN ≥ 6) shows a slight decrease as the average grain diameter decreases to 5.60 nm, which is caused by the less compression in the deformation region. Furthermore, fluctuation in the number of over-coordinated atoms when cutting on the polycrystal workpiece is much more obvious, since the formation of the over-coordinated atoms is influenced by microstructure characteristics like GBs and crystal orientations.

#### 3.2.3. Internal Stress and Temperature

**Figure 12:**
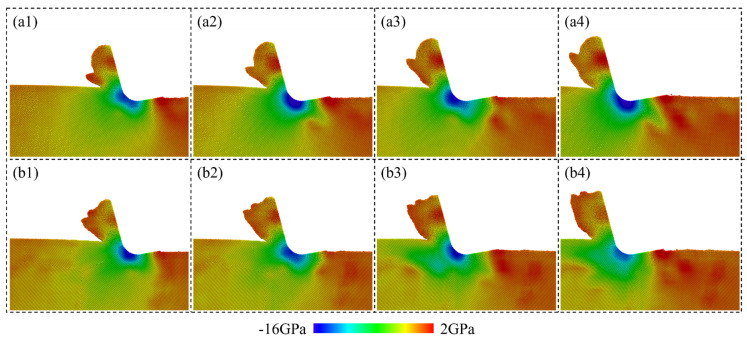
Hydrostatic stress distribution in a workpiece during cutting on: (**a1**–**a4**) Single-crystal workpiece. (**b1**–**b4**) Polycrystal workpiece with average grain diameter of 5.60 nm.

**Figure 13:**
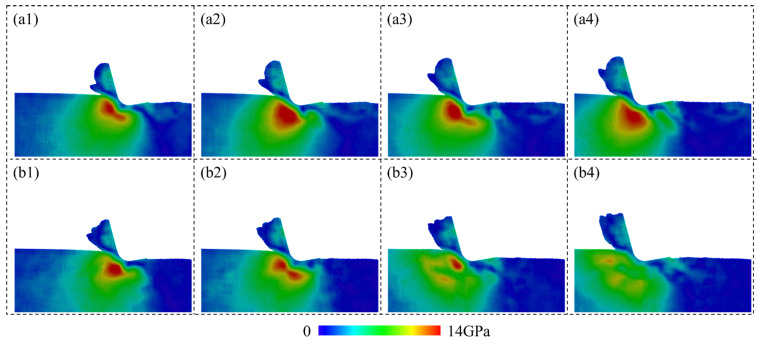
von Mises stress distribution in a workpiece during cutting on: (**a1**–**a4**) Single-crystal workpiece. (**b1**–**b4**) Polycrystal workpiece with average grain diameter of 5.60 nm.

**Figure 14:**
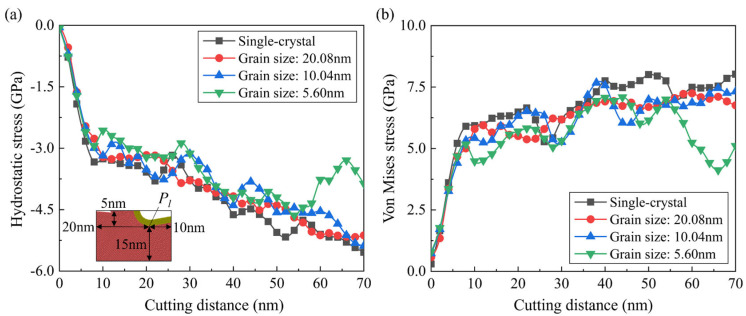
Variation in the averaged (**a**) compressive stress and (**b**) von Mises stress in the deformation region during the cutting process.

To further explore the grain size effect on subsurface deformation, the hydrostatic stress *σ_h_* and von Mises stress *σ_v_* of the workpiece atoms were calculated via [45]:

(2) $$\sigma_{h}=\frac{{(\sigma }_{\mathrm{xx}}+\sigma_{\mathrm{yy}}+\sigma_{\mathrm{zz}})}{3}$$

(3) $$\sigma_{v}=\sqrt{\frac{\;(\sigma_{\mathrm{xx}}-\sigma_{\mathrm{yy}}{)}^{2}+(\sigma_{\mathrm{yy}}-\sigma_{\mathrm{zz}}{)}^{2}+(\sigma_{\mathrm{zz}}-\sigma_{\mathrm{xx}}{)}^{2}\;+6(\tau_{\mathrm{xy}}^{2}+\tau_{\mathrm{yz}}^{2}+\tau_{\mathrm{zx}}^{2})}{2}}$$

where *σ_xx_*, *σ_yy_*, *σ_zz_*, *τ_xy_*, *τ_xz_*, and *τ_yz_* are stress tensors in the Cartesian coordinate system. Figure 12 shows the calculated hydrostatic stress of Si atoms when cutting on a single-crystal workpiece and polycrystal workpiece with average grain diameter of 5.60 nm. For single crystals, a high compressive region is formed near the cutting tool edge, indicating the HPPT of workpiece material in the deformation region. Obvious concentration of the tensile stress is observed on the machined surface, which is caused by the tearing off effect from the cutting tool. For brittle materials like Si, concentration of the tensile stress is an important source for the surface tearing, which is disadvantageous for high-quality surface fabrication. When cutting on a polycrystal workpiece, fluctuation in the compressive stress is more apparent, as grains and GBs could cause an unstable material removal process and release the compression in the deformation region by slip along GBs. In addition to the hydrostatic stress, concentration of the von Mises stress is detected near the shear band ahead of the cutting tool when cutting on the single-crystal workpiece, as shown in Figure 13(a1–a4). Periodic variation in the stress can be observed due to the regular accumulation and release of the material load during the cutting process. While for the polycrystal workpiece, the shear deformation is greatly determined by the crystal grains and boundaries. Therefore, irregular variation in the von Mises stress is observed during the cutting process. Furthermore, variation in the average hydrostatic and von Mises stress in the deformation region during cutting is calculated, as shown in Figure 14. The deformation region is defined as a box that covers the main contact region between tool and workpiece regarding the lowest point of cutting tool *P_l_*, as shown in Figure 14a. It is observed that the compressive stress and von Mises stress both decrease as the average grain size decreases. When cutting on the polycrystal workpiece, the stress concentration inside the workpiece is decreased as the GBs facilitate sliding of the grains and squeeze of the disordered atoms, which release the strain energy in the deformation region. Due to the slip motion and deformation of grains with different orientations, fluctuation in average stress becomes much more apparent as the grain size decreases.

**Figure 15:**
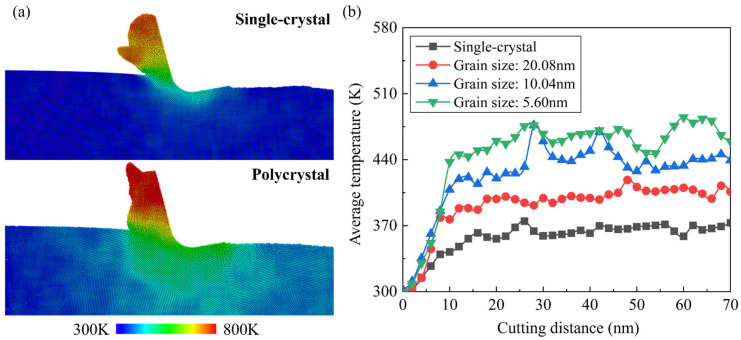
Workpiece temperature in the cutting simulation. (**a**) Temperature distribution of single-crystal workpiece and polycrystal workpiece with average grain diameter of 5.60 nm. (**b**) Variation of the average temperature of workpiece atoms during the cutting process.

The workpiece temperature is calculated by:

(4) $$T=\frac{\sum_{i}{\mathrm{mv}}_{i}^{2}}{3Nk_{B}}$$

where *N* is the number of atoms, *v_i_* represents the velocity of the *i*th atom, and *k_B_* is the Boltzmann constant (1.3806503 × 10^−23^ J/K). Snapshots of the temperature distribution of single-crystal and polycrystal workpieces during cutting are shown in Figure 15a. In MD simulation of nanometric cutting, the generated heat is mainly dissipated by chips and the thermostat layer in workpiece. Therefore, the highest temperature of the workpiece is observed in chips. It can be observed that the temperature of the polycrystal workpiece is higher than single crystals, which can be attributed to the hindered heat transfer by GBs inside the workpiece. Figure 15b shows the variation in average workpiece temperature during cutting. Obvious fluctuation in average temperature can be observed when cutting on the polycrystal workpiece, especially with smaller grains. When cutting on the polycrystal workpiece, the unstable material removal induced by GBs could affect the friction behavior and energy dissipation process, which determines the variation in temperature in the deformation region.

## 4. Conclusions

In the present study, MD simulation was conducted to explore the machining mechanism of polycrystalline Si. The effect of grain size on material removal behavior and subsurface damage evolution was discussed. The major conclusions are summarized as follows.

1. In nanometric cutting, the stagnation region can be less apparent and more nanogrooves are generated at GBs when cutting on the polycrystal workpiece. As the grain size decreases, the material removal process becomes unstable and higher removal efficiency is achieved. Massive nanocrystals are generated in the workpiece due to the intra-granular facture and squeeze of the disordered atoms, while on the machined surface, the elastic recovery layer becomes irregular and pits are observed on the machined surface.
2. When cutting on the polycrystal workpiece, the average frictional coefficient is higher than single crystals and increases as the grain size decreases. This variation is attributed to the decrease in nominal force, since the density of GBs tends to have a more apparent influence in releasing the downward compression than resistance of cutting tool motion.
3. For a polycrystal workpiece, slip along the GBs merges as a nonnegligible process of the plastic deformation in workpiece. With the decrease in grain size, the elastic deformation ahead of the cutting tool can be less apparent as the strain energy is dissipated by enhanced slip motion. More 5-coordinated atoms are generated, especially when the grain size decreases, while fewer atoms with high coordination numbers (CN ≥ 6) are generated in the deformation region.
4. When cutting on the polycrystal workpiece, the fluctuation in compressive stress and von Mises stress is more apparent and the workpiece temperature is higher than with a single-crystal workpiece. As the grain size decreases, the average stress decreases, since the GBs facilitate plastic deformation and release the strain energy while the average workpiece temperature increases due to the impediment of heat transfer by GBs.

## Figures and Tables

**Table 1:** Parameters of the MD simulation model.

Properties	Parameters
Size of workpiece (*x* × *y* × *z*)	100.0 nm × 5.4 nm × 44.5 nm
Rake/flank angle of cutting tool	−10°/10°
Total number of atoms	About 1.33–1.34 million
Cutting temperature	300 K
Material removal thickness	5 nm
Radius of tool edge	5 nm
Cutting speed	20 m/s

## Data Availability

The original contributions presented in the study are included in the article. Further inquiries can be directed to the corresponding author.
